# Human menstrual blood-derived stem cells promote functional recovery in a rat spinal cord hemisection model

**DOI:** 10.1038/s41419-018-0847-8

**Published:** 2018-08-29

**Authors:** Qinfeng Wu, Qinghua Wang, Zhangjie Li, Xiangzhe Li, Jing Zang, Zhangwei Wang, Chen Xu, Yujia Gong, Jiaqi Cheng, Haoming Li, Guangyu Shen, Chuanming Dong

**Affiliations:** 1 Department of Anatomy, Medical School of Nantong University, Laboratory Animal Center of Nantong University, Nantong, Jiangsu Province 226001 China; 2 Department of Rehabilitation Medicine, Suzhou Hospital affiliated to Nanjing Medical University, Suzhou Science & Technology Town Hospital, 215153 Suzhou, Jiangsu Province China; 3 Department of Rehabilitation Medicine, Affiliated Hospital of Nantong University, 226001 Nantong, Jiangsu Province China; 4 Department of Rehabilitation Medicine, Zhangjiagang First People’s Hospital, 215600 Zhangjiagang, Jiangsu Province China

## Abstract

Spinal cord injury (SCI) is associated with a dismal prognosis including severe voluntary motor and sensory deficits in the presence of the current therapies, thus new and efficient treatment strategies are desperately required. Along with several advantages, such as easy accessibility, high-yield, potential of enormous proliferation, menstrual blood-derived mesenchymal stem cells (MenSCs) have been proposed as a promising strategy in regeneration medicine. In this study, the MenSCs were transplanted into incomplete thoracic (T10) spinal cord injury (SCI) rats, all rats were sacrificed at 7, 14, and 28 days after surgery. Based on the results, we found that MenSCs transplantation improved the hind limb motor function. Besides, H&E staining showed that MenSCs treatment markedly reduced cavity formation in the lesion site. Furthermore, treatment by MenSCs showed more MAP2-positive mature neurons, as well as axonal regeneration manifested by NF-200 and less expression of chondroitin sulfate proteoglycans (CSPGs) than the non-treatment in the lesion site. Additionally, immunofluorescence, Western blot, and qRT-PCR methods showed that levels of brain-derived neurotrophic factor (BDNF) were significantly higher in the injured spinal cord after implantation of MenSCs. Results of qRT-PCR indicated that inflammatory factors, including TNF-α and IL-1β were inhibited after MenSCs transplantation. The improved motor function of hind limb and the increased cell body area of motor neurons were suppressed by blocking of the BDNF-TrkB signaling. It was eventually revealed that MenSCs implantation had beneficial therapeutic effects on the rehabilitation of the rat spinal cord hemisection model, mainly by enhancing the expression of BDNF. MenSCs transplantation may provide a novel therapeutic strategy for patients with SCI in the future.

## Introduction

Spinal cord injury (SCI), leading to permanent sensory and motor function deficits below the lesion level due to the lack of regeneration of the damaged axons, and has been remained as one of the hot clinical challenges^1,2^. The main threats of SCI recovery include the drastic loss of neurons, formation of cystic cavity and glial scar, abatement of neurotrophic factor, and accumulation of myelin-associated inhibitor^3,4^. The current therapy of SCI involves reduction of swelling with corticosteroid drug, relieving excess pressure on the spinal cord by surgery, stabilization of the spine, rehabilitation training, pain relief, and prevention and treatment of complications^5^. However, the self-repair ability of central nervous system is extremely limited, reflecting that these traditional therapies cannot substantially restore neurological function in the injured spinal cord.

Nowadays, stem cell therapy has become an effective strategy for SCI^6,7^. The most promising stem cell is neural stem cells (NSCs), because NSCs harbor a certain ability to differentiate into neural and glial cells when are transplanted in the site of SCI^8^. However, some drawbacks also shadowed with NSCs: adult NSCs were not available for autologous cell transplant, fetal-derived NSCs had ethical concerns, and a previous study reported a boy with ataxia telangiectasia (AT), who received human fetal neural stem cell transplantation, while he suffered from brain tumor^9^.

Mesenchymal stem cells (MSCs) are multipotent adult stem cells which can differentiate into multiple cell types^10^. MSCs can be isolated from the bone marrow, umbilical cord blood, adipose tissue, muscle, and dental pulp. In comparison with NSCs, MSCs show a high degree of genomic stability during culture and typically do not result in tumor formation. Transplantation of MSCs into the injured rat spinal cord promoted tissue preservation by directly replacing the damaged cells, decreasing the cyst and injury area, stimulating axonal sprouting, producing neurotrophic factors, as well as inhibiting inflammatory cytokines^11,12^.

However, the use of these MSCs involves a number of barriers. Human umbilical cord is limited to collection at birth. Bone marrow and fat biopsy are painful and requires general anesthesia. Thus, much more accessible source of certain multipotent stem cells should be found for substitution in clinical studies, which are easily available and feasible.

Menstrual blood-derived mesenchymal stem cells (MenSCs) were firstly discovered by Meng et al. in 2007 from menstrual fluids, as a novel source of MSCs^13^. With the potential of multi-directional differentiation, MenSCs are able to undergo adipogenic, chondrogenic, osteogenic, myogenic, and neurogenic differentiation in vitro^14,15^. Compared with other sources of MSCs, MenSCs can be easily selected in a periodic manner and obtained by a noninvasive method, avoiding the ethical issues^16^, do not form teratomas^17^, and can be expanded by at least 20 passages without genetic abnormalities^18^. In recently studies, MenSCs showed potential therapeutic applications in a variety of animal disease models, such as stroke^19^, type 1 diabetes^20,21^, myocardial infarction^22^ and so on.

In the present study, MenSCs were implanted into the rat spinal cord lesion site after hemisection of T10 spinal cord segment. After 4 weeks study on locomotor recovery and microenvironment in the injury site, our results demonstrated that MenSCs promoted axon growth, reduced glial scar and improved microenvironment. The findings in this observation may provide useful experimental evidences of the clinical value of MenSCs transplantation for SCI recovery.

## Results

### Isolation and identification of MenSCs

**Fig. 1:**
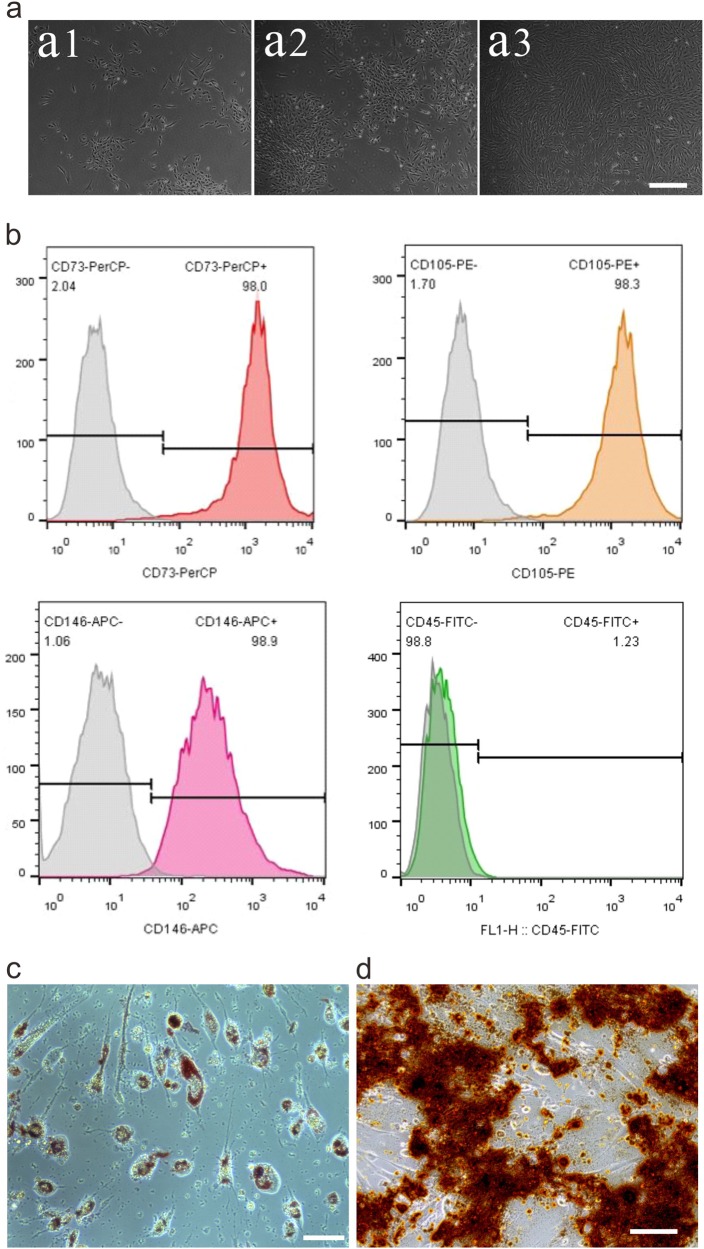
Characterization of menstrual blood-derived stem cells. **a** The morphology of cultured MenSCs. **a1** The shape of cultured MenSCs at day 4. **a2** The isolated MenSCs showed appearance of initial colony forming unit after 7 days culture. **a3** MenSCs reached 85% confluence at passage 2. **b** FACS was used to profile the surface antigens of MenSCs, MenSCs are positive for CD73, CD105, and CD146, negative for CD45. **c** Alizarin red staining was taken to check the osteogenic differentiation of MenSCs after 28 days induction. **d** Oil red O staining was taken to check the adipogenesis ability of MenSCs after 21 days differentiation. Scale bar: 100 μm

The isolated human MenSCs grew in attachment with the dish, and exhibited spindle-like shape as fibroblasts with round nucleus in the middle of the cell (Fig. 1a). To confirm the characters of MenSCs, FACS analysis was carried out to investigate the mesenchymal-specific markers. The data showed that MenSCs were positive for CD73 (98.0%), CD105 (98.3%), and CD146 (98.9%), but negative for CD45 (1.23%) (Fig. 1b). In addition, the MenSCs can be induced into osteoblast cells confirmed by Alizarin red S staining and adipocytes confirmed by Oil Red O staining (Fig. 1c, d).

### The influences of MenSCs on treatment of SCI

**Fig. 2:**
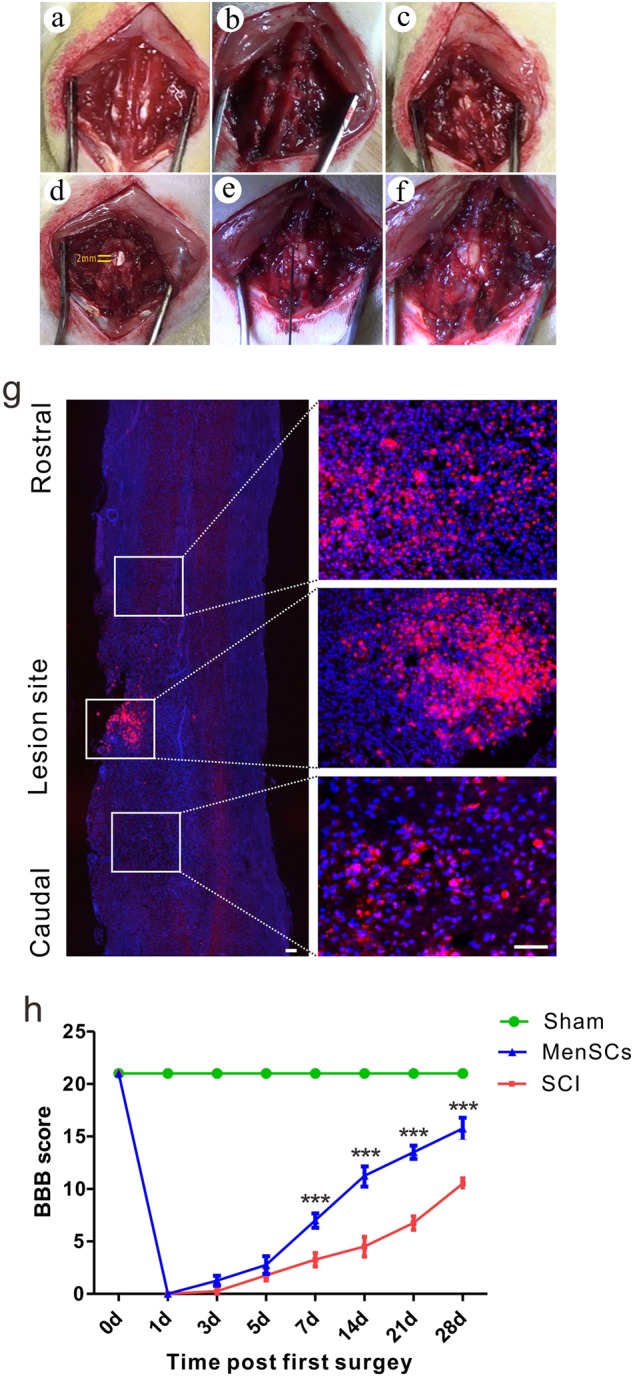
MenSCs survived and improve the locomotion of SCI rat. **a–f** The sequential surgery procedures of T10 spinal cord hemisection model and implantation of MenSCs into the lesion site. **g** Immunofluorescence detection of transplanted MenSCs labeled with CM-Dil (red) in caudal, rostral and lesion sites of injured spinal cord. Scale bar: 100 μm. **h** Greater improvement in the motor function of the hindlimb was observed in the MenSCs group, compared with the SCI group from day 7 to day 28 post surgery. ***P < 0.001. Two-way ANOVA repeated measurement followed by Bonferroni’ post hoc test

To clarify the function of MenSCs in treatment of SCI, spinal cord lateral hemisection was conducted over the 10th thoracic vertebral segment to construct a SCI rat model. The sequential procedures were demonstrated as skin incision, exposure of spinal cord, boning vertebral column, and laminectomy. For this purpose, a 2 mm of spinal cord was fully removed by micro-scissors, MenSCs injection, and wound closure (Fig. 2a–f). In addition, the Dil-labeled MenSCs were checked by immunofluorescence in the caudal, rostral, and lesion site of the injured spinal cord. Much more abundant fluorescence was observed in the lesion site and less in the caudal and rostral sites (Fig. 2g). Moreover, Basso, Beattie, and Bresnahan (BBB) scores were taken into account to evaluate the locomotor function of the hind limbs. Analyses of the BBB score up to 28 days postinjury revealed that MenSCs transplantation improved the locomotor function of the rats significantly (two-way ANOVA repeated measurement, *F*_(2,6)_ = 1026, *P* < 0.0001; Fig. 2h). Furthermore, Bonferroni posttests demonstrated that the rats in MenSCs group acquired a significant benefit in locomotor ability than the SCI group from 7 to 28 days after surgery (*P* < 0.001; Fig. 2h).

### Improving the integrity of injured site and decreasing the volume of lesion cavities by MenSCs treatment method

**Fig. 3:**
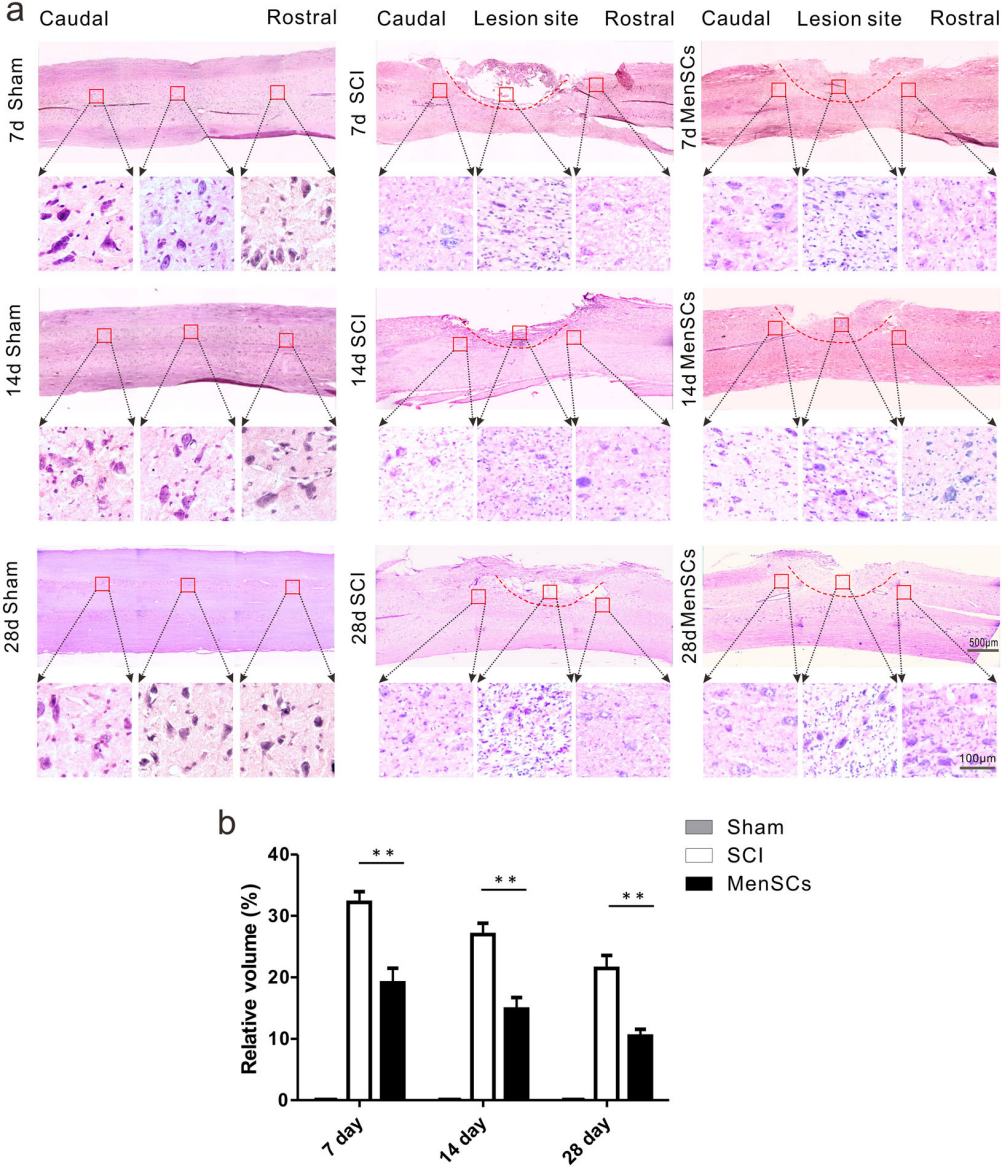
MenSCs enhanced the recovery of the injured cord. **a** H&E staining was adopted for histological analysis. The left column is the sham group, the middle column is the SCI group, and the right column shows the MenSCs group. The recovery process shows in time course at 7, 14, and 28 days after surgery in the vertical direction. The upper panel displays the low magnification of horizontal spinal cord sections; the lower panel indicates the higher magnification of the rostral, lesion, and caudal site of the injured spinal cord. The dashed lines indicate host/lesion interfaces. **b** This graph shows relative cavity formation in the sagittal section of spinal cord among the three groups. The relative cavity sizes were calculated by dividing the cavity volume by the spinal cord volume at the lesion site. There were significant differences between SCI and MenSCs groups. **P < 0.01. Two-way ANOVA repeated measurement followed by Bonferroni’ post hoc test

To figure out the delicate variations during the recovery process, histological sections were undergone in hematoxylin and eosin (H&E) staining at time points of 7th, 14th, and 28th days of post-surgery. It was revealed that there were inflammatory cell infiltration, nerve cell shrinkage, and vacuolization in the control group. While MenSCs treatment strategy could reduce the inflammatory cell infiltration and vacuolization, which method improved the integrity of the tissue not only in the lesion site, but also in the rostral and caudal sites (Fig. 3a). Additionally, the cavity volume at the lesion site was measured in H&E sections. The cavity volume, as a percentage of the spinal cord volume at the lesion site, was calculated for each group. The values were 32.2 ± 1.75% and 19.1 ± 2.36% in the SCI and MenSCs groups, respectively at 7 days after surgery, followed by 26.9 ± 1.86% and 14.9 ± 1.84% in the SCI and MenSCs groups, respectively, at 14 days after operation. This trend was proceeded by 21.4 ± 2.15% and 10.4 ± 1.13% in the SCI and MenSCs groups, respectively, at 28 days after surgery (Fig. 3b). There was a significant difference between SCI and MenSCs groups, indicating that MenSCs transplantation has markedly reduced cavity formation in the lesion site (*P* < 0.01).

### Increasing the survival of neuron cells in the lesion site by MenSCs treatment

**Fig. 4:**
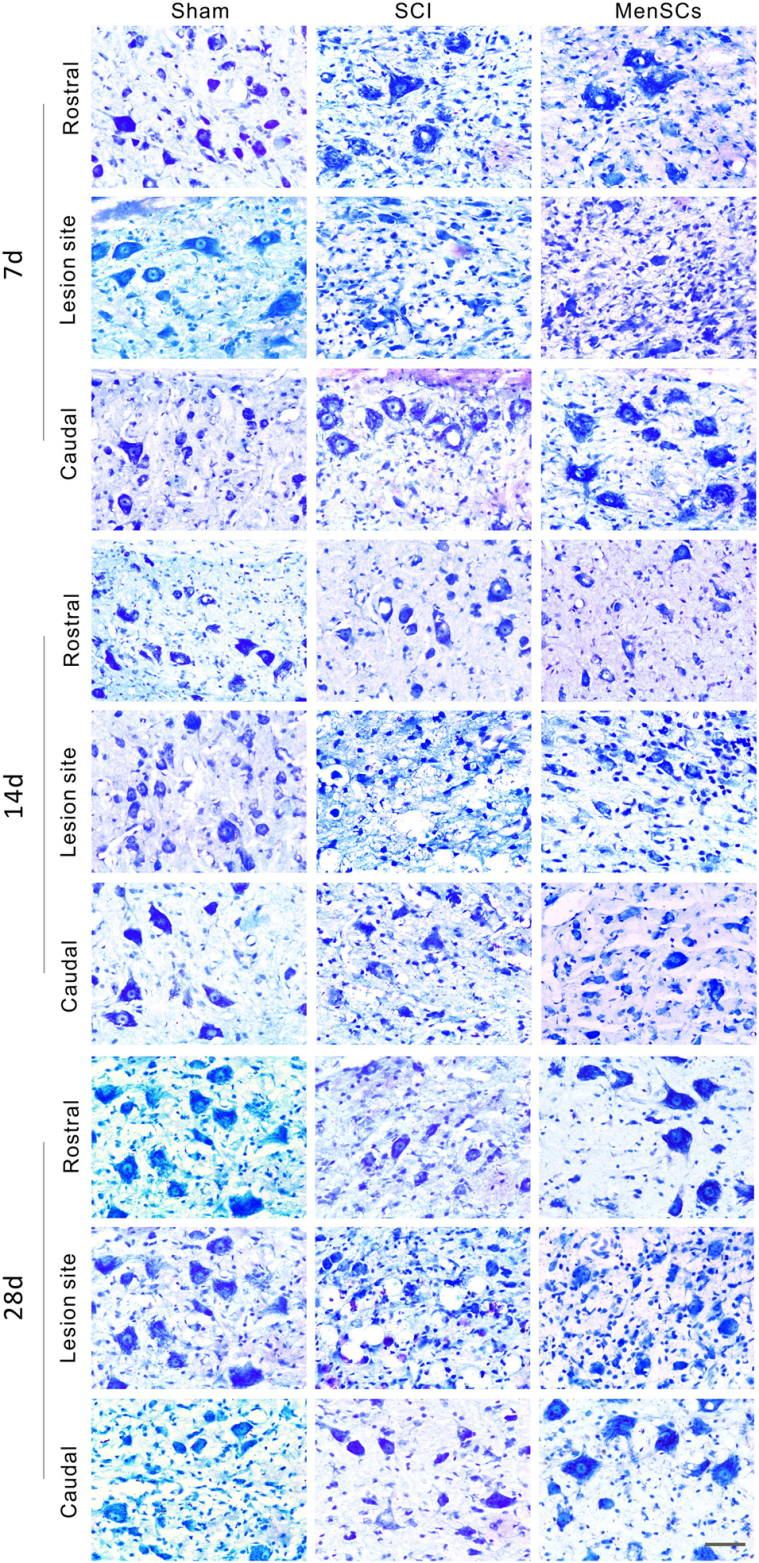
MenSCs increased viability of neurons in injured spinal cord. In the Nissl staining picture, the three panels/blocks which contains 3 × 3 pictures exhibit sections at 7, 14, and 28 days after surgery. Sham group in the left column, SCI group in the middle, and MenSCs group in the right column. The fields of rostral, lesion, and caudal site are listed from top to bottom in each panel/block. Scale bar: 50 μm

To detect the viability of the neurons during the recovery process after SCI, Nissl staining was adopted to reveal the morphology and numbers of Nissl bodies in rostral, lesion, and caudal sites in both SCI and MenSCs groups at 7, 14, and 28 days after surgery. In the SCI group, most of Nissl bodies were dissolved and disappeared, which surrounded by inflammatory cell infiltration at 7th day. Then, a large number of vacuoles were appeared at 14th and 28th day. The number of Nissl bodies in MenSCs group was much higher than that of in SCI group at each time point and site, especially at lesion sites (Fig. 4).

**Fig. 5:**
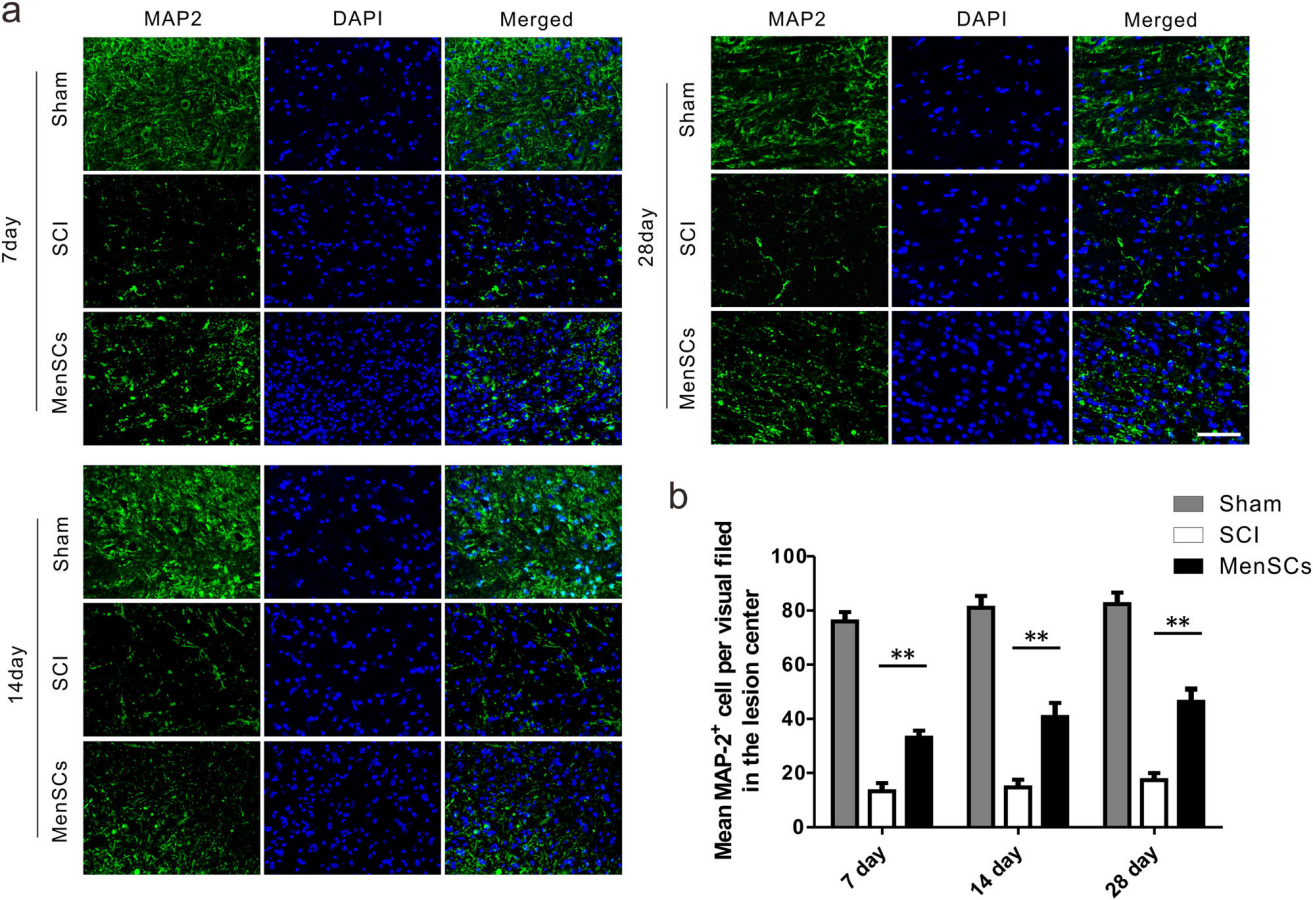
MenSCs treatment increased the survival of neuron cells in the lesion site. **a** Immunofluorescence observation was used to reveal the expression of MAP2 among the three groups at 7, 14, and 28 days after surgery. **b** Quantification of Map2-positive cells in the lesion center in each group at day 7, 14, and 28 after SCI, respectively. **P < 0.01. Two-way ANOVA repeated measurement followed by Bonferroni’ post hoc test. Scale bar: 100 μm

Furthermore, rats were sacrificed and spinal cord sections were assessed by immunohistochemistry at 7, 14, and 28 days after MenSCs implantation. Immunostaining results showed that there were higher numbers of MAP2-labeled mature neurons in the lesion area at the MenSCs group in comparison with the SCI group (Fig. 5a). The mean MAP2^+^ cell per visual field in the lesion center was 76.0 ± 3.46, 13.3 ± 2.96, and 33.0 ± 2.65 in Sham, SCI, and MenSCs groups, respectively, at 7 days after surgery, 81.0 ± 4.36, 14.7 ± 2.90, and 40.7 ± 5.20 in Sham, SCI, and MenSCs groups, respectively, at 14 days after operation. Besides, that value was 82.3 ± 4.26, 17.3 ± 2.60, and 46.3 ± 4.67 in Sham, SCI, and MenSCs groups, respectively, at 28 days after surgery (Fig. 5b). The quantitative results revealed a significant increase in MAP2-positive mature neurons in the MenSCs group compared to the SCI group (*P* < 0.01).

### Promoting axonal regeneration in the lesion site by MenSCs treatment strategy

**Fig. 6:**
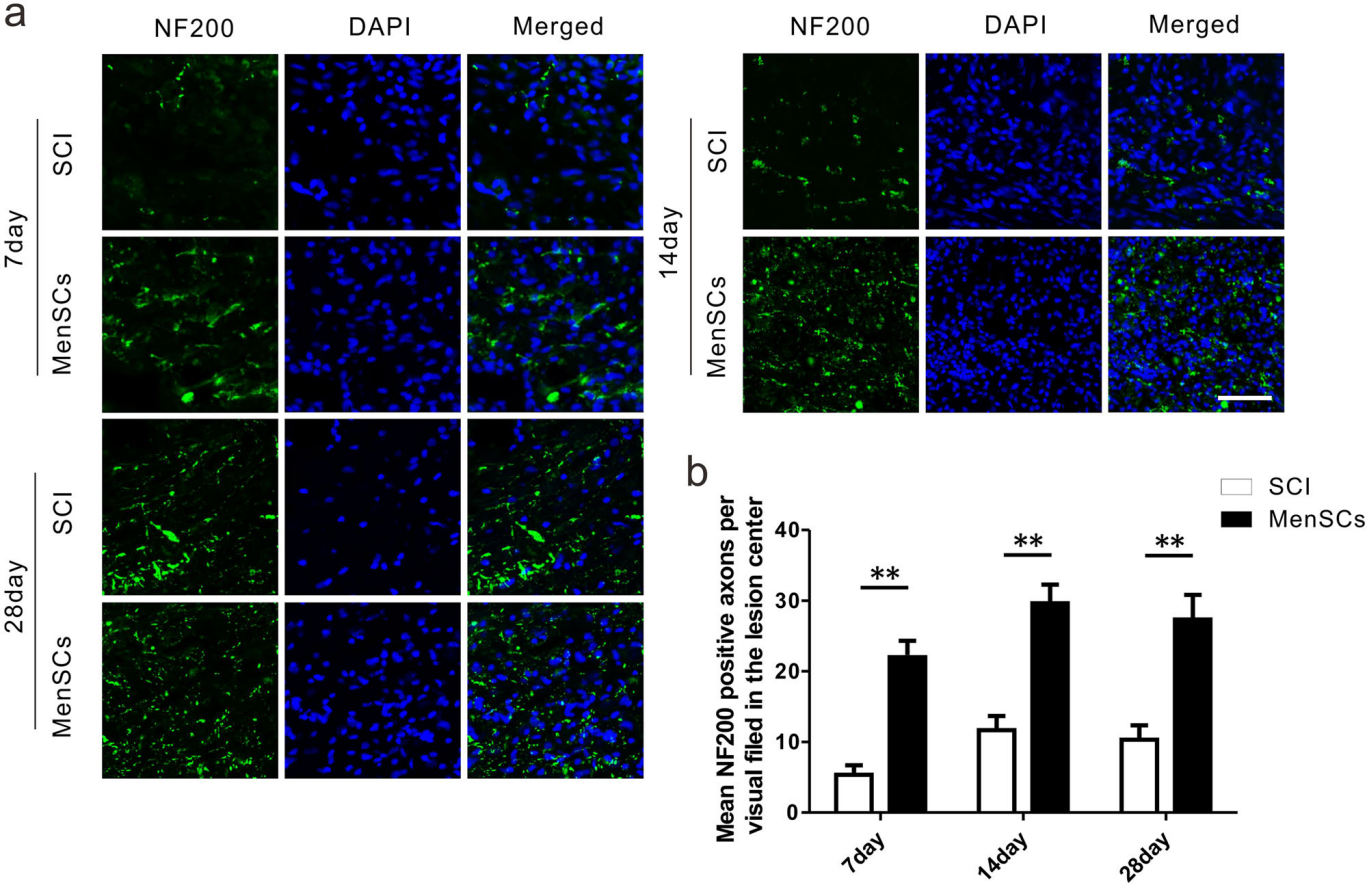
MenSCs increased axonal fibers in hemisected spinal cord. **a** NF200-positive axons cells in the lesion site were checked in both SCI and MenSCs groups at 7, 14, and 28 days after surgery. **b** Quantification of NF200-positive axons in the lesion center in each group at 7, 14, and 28 days after surgery, respectively. **P < 0.01. Two-way ANOVA repeated measurement followed by Bonferroni’ post hoc test. Scale bar: 100 μm

Neurofilament is one of the main components of the neuronal cytoskeleton, which is well-known for providing structural support for the axon. We observed the effect of MenSCs treatment on the axonal sprouting after SCI by immunostaining for NF200. At 7 days after SCI, the immunostaining results showed that there were more NF200-positive axonal fibers, which were widely distributed in the lesion site, in the MenSCs group than the SCI group (Fig. 6a). The mean NF200-positive axons per visual filed in the lesion center was 5.3 ± 0.88 and 22.0 ± 2.30 in SCI and MenSCs groups, respectively, at 7 days after surgery, 11.7 ± 2.03 and 29.7 ± 2.60 in SCI and MenSCs groups, respectively, at 14 days after operation. Besides, that value was 10.3 ± 2.03 and 27.3 ± 3.48 in SCI and MenSCs groups, respectively, at 28 days after surgery (Fig. 6b). The quantitative results revealed that the mean NF200-positive axons per visual field in MenSCs group were much greater than SCI group in the lesion center. At 14th and 28th day, the percentage of NF200-positive cells still kept the same tendency as that at 7 days (*P* < 0.01).

### Reducing the formation of secondary glial scar by MenSCs treatment strategy

**Fig. 7:**
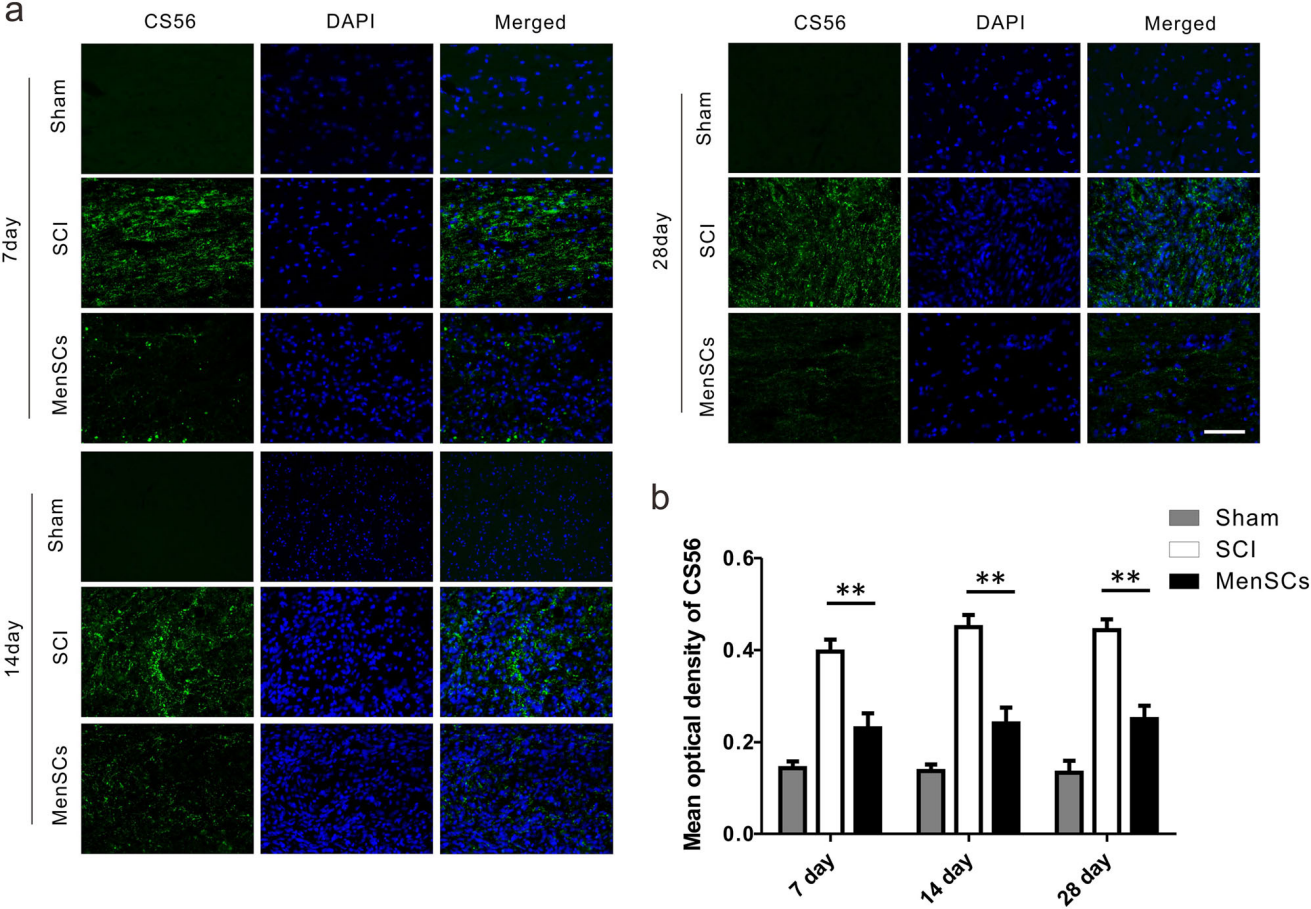
MenSCs transplantation reduced secondary glial scarring after T10 spinal cord hemisection. **a** Immunofluorescence images showing CS56^+^ glial scar in the lesion site at each time point in each group. **b** Quantification of the mean optical density of CS56. There were significant differences at 7, 14, and 28 days following surgery in the MenSCs group compared with that in the SCI group.**P < 0.01. Two-way ANOVA repeated measurement followed by Bonferroni’ post hoc test. Scale bar: 100 μm

The formation of glial scar after SCI is an important factor that affects the regeneration of neuron and nerve fiber. Previous studies have demonstrated that chondroitin sulfate proteoglycans (CSPGs) play a significant role in glial scar formation, inhibiting axon regeneration after SCI. The CS56 antibody has been reported to be specific for the glycosaminoglycan (GAG) portion of native CSPGs. At 7 days after SCI, longitudinal sections of the spinal cord containing the lesion areas were used to measure glial scar formation labeled by CS56. We found that the CS56 signal is stronger in the SCI group, in comparison with that of in the MenSCs group. At 14th and 28th day, expression of CS56 was kept as same as the 7th day (Fig. 7a). The quantitative results revealed that the mean optical density (MOD) in the SCI group (0.397 ± 0.026) was significantly higher than that in the MenSCs group (0.230 ± 0.032) at 7 days after surgery, 0.450 ± 0.026 and 0.240 ± 0.035 in SCI and MenSCs groups, respectively, at 14 days after operation. Besides, that value was 0.443 ± 0.023 and 0.250 ± 0.029 in SCI and MenSCs groups, respectively, at 28 days after surgery (Fig. 6b) (*P* < 0.01).

### Transplantation of MenSCs enhanced the expression of brain-derived neurotrophic factor (BDNF) and decreased the expression of TNF-α and IL-1β in the lesion site after SCI

**Fig. 8:**
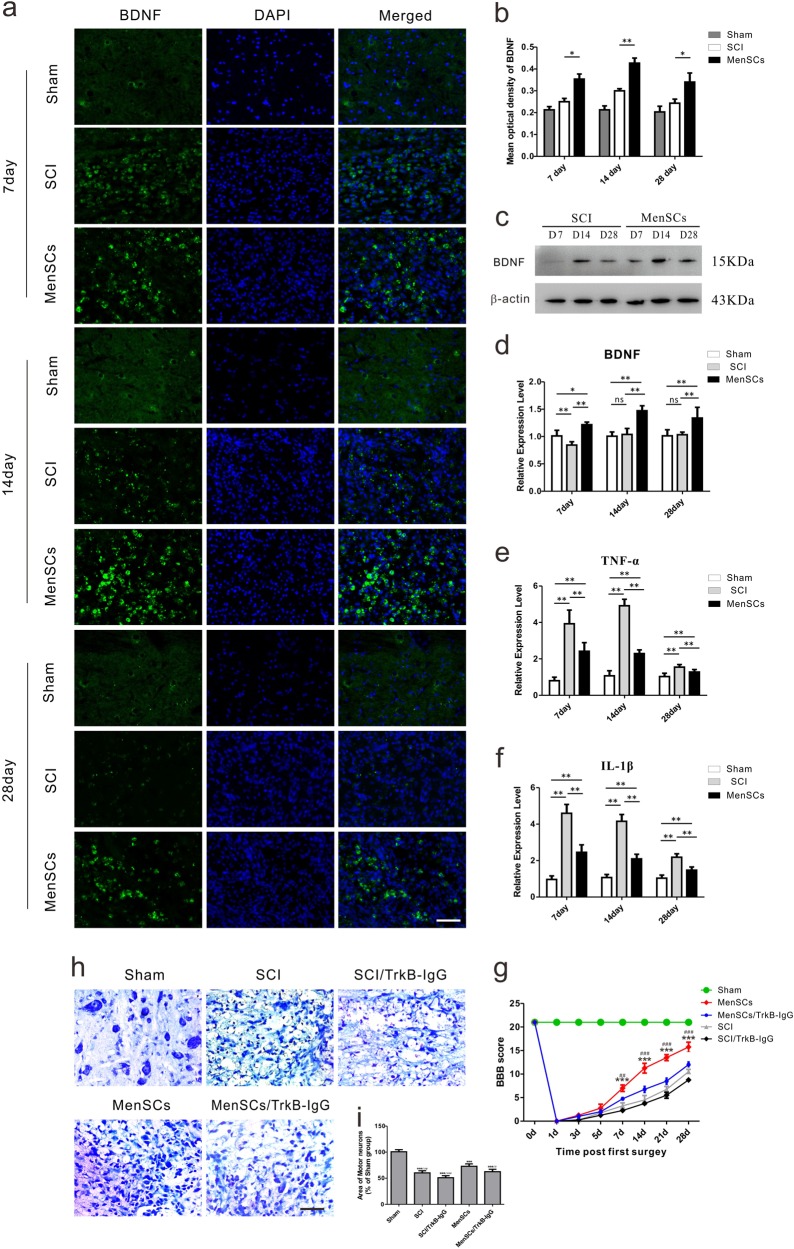
MenSCs implantation enhanced the expression of BDNF and decreased the expression of TNF-α and IL-1β. **a** The expression of BDNF in the lesion site was determined by immunofluorescence staining in each group at 7, 14, and 28 days after surgery. Scale bars, 100 μm. **b** Shows the mean optical density of BDNF. **c** Western blot analysis of BDNF expression in the lesion site between MenSCs group and SCI group at indicated time points after SCI. **d** qRT-PCR analysis of BDNF expression in the lesion site at indicated time points after SCI. **e** qRT-PCR analysis on the expression of TNF-α at every time point after SCI. **f** qRT-PCR analysis on the expression of IL-1β at every time point after SCI. *P < 0.05, **P < 0.01, ns: no significance. Scale bar: 100 μm. **g** TrkB-IgG was used to block the BDNF-TrkB signaling. The results showed that MenSCs treatment could promote the recovery of hindlimb motor function in SCI rats. However, the promotion effect of locomotor function was inhibited significantly by the blockade of BDNF-TrkB signaling. ^##^*P* < 0.01, ^###^*P* < 0.001 compared with the MenSCs/TrkB-lgG group; ***P < 0.001 compared with the SCI group. Two-way ANOVA repeated measurement followed by Bonferroni’ post hoc test. **h** The Nissl staining of motor neurons in the lesion site of the spinal cord at 14 days after surgery. **i** The statistical graph of the cell body area of motor neurons in the spinal cord. Compared with Sham group, ***P < 0.001; Compared with MenSCs group, ^#^*P* < 0.05, ^##^*P* < 0.01, ^###^*P* < 0.001. Scale bar: 50 µm

Histological analysis of the BDNF expression in the lesion site showed that BDNF fluorescence intensity in the MenSCs group is higher than that of in the SCI group at 7, 14, and 28 days after SCI (Fig. 8a). In the MenSCs group, the fluorescence intensity of BDNF significantly increased at 7 days after injury, and was maximal at 14th day, then gradually decreased at 28th day (Fig. 8b). The quantitative results revealed that the MOD in the MenSCs group (0.353 ± 0.023) was significantly higher than that in the SCI group (0.250 ± 0.015) at 7 days after surgery, 0.427 ± 0.023 and 0.300 ± 0.010 in MenSCs and SCI groups, respectively, at 14 days after operation. Besides, that value was 0.340 ± 0.042 and 0.243 ± 0.019 in MenSCs and SCI groups, respectively, at 28 days after surgery. (*P < 0.05 or **P < 0.01). Additionally, the results of Western blot and qRT-PCR analyses also confirmed that MenSCs increased the expression levels of BDNF in the lesion site after SCI (Fig. 8c,d). Besides, qRT-PCR analysis showed that the expression of two inflammatory factors, i.e. tumor necrosis factor-alpha (TNF-α) and interleukin-1β (IL-1β), was suppressed after MenSCs transplantation in each group at the mentioned time points after SCI (Fig. 8e, f).

### Blocking of BDNF-TrkB signaling inhibits the promotion effect of motor function recovery after MenSCs transplantation in SCI rats

TrkB-IgG was used to block the BDNF-TrkB signaling, and BBB scale was used to observe the recovery of hindlimb motor function after MenSCs transplantation in SCI rats. The results showed that MenSCs treatment could promote the recovery of hindlimb motor function in SCI rats. However, the promotion effect of locomotor function was inhibited significantly by the blockade of BDNF-TrkB signaling (Fig. 8g). Nissl staining was performed to observe the cell body area and morphologic characteristics of motor neurons in lesion sites in Sham, SCI, SCI/TrkB-IgG, MenSCs, and MenSCs/TrkB-IgG groups at 14 days after surgery (Fig. 8h). The cell body area of motor neurons in the MenSCs/TrkB-IgG group was significantly smaller than that in the MenSCs group (72.95 ± 3.986% in the MenSCs group and 62.51 ± 4.271% in the MenSCs/TrkB-IgG group, relative to the Sham group, respectively, *P* < 0.05) (Fig. 8i). Collectively, results above demonstrated that BDNF-TrkB signaling was vital for promoting locomotor recovery of SCI rats.

## Discussion

Pioneering work with different stem cells has acquired the precedent and the recent advances in the treatment of SCI. With the ability to differentiate into neuronal and glial cells, the MSCs are quite appropriate for SCI therapy^23^. Nowadays, application of MSCs in treating SCI has been reported by plenty of studies, which may promote the recovery of motor function after SCI^24–26^.

Menstrual blood collected in each period contains the shed pieces of the endometrium in the functionalis layer, representing a source of endometrial MSCs^27^. In comparison with bone marrow mesenchymal stem cells (BMSCs), MenSCs exhibitd more superiorities, which lies in the higher rate of proliferation as well as self-renewal capacity^28^. In addition, the expression of TuJ1, ChAT, and Pax6 in the differentiated cells of MenSCs was higher than that in BMSCs^29^.

To our knowledge, the MenSCs strategy is used for the first-time in the treatment of SCI in rat. We firstly identified the characteristics of MenSCs used in this study. The biomarkers of MenSCs were checked by FACs and demonstrated a similar pattern with other studies^30^. In the present study, as shown in Fig. 2a–f, rats underwent T10 spinal cord hemisection by removing 2 mm of the spinal cord. The BBB score was taken into account to affirm the improvement of locomotion after MenSCs transplantation, in which a greater enhancement of locomotor recovery was observed in the MenSCs group than that of in SCI group ranged from 7 to 28 days after MenSCs transplantation. Pathological staining disclosed that MenSCs transplantation drastically decreased the size of the necrotic cavities, increased the survival rate of neurons in the lesion site and preserved more tissue as well. The above-mentioned results showed the possibility of MenSCs as a useful strategy to treat SCI.

Generally, stem cells affect the spinal cord repair course by regulating neuron viability and survival, maintaining favorable niche, suppressing inflammatory reaction, secreting certain neurotrophic factors and recruiting endogenous progenitors to in situ replenish the loss of apoptotic cells^31,32^. In the present study, we investigated the expression of important neuronal and glial scar markers at different time points after MenSCs transplantation. NF-200 mainly existed in mature axon, MAP-2 enriched in the dendrites, which was considered as a maker of mature neuron^33^. The acquired data by this study showed that the expressions of NF-200 and MAP-2 were markedly increased from 7 days after cell transplantation. Belonging to the inhibitory molecules family produced by glial scar tissue, and CSPGs are taken as mechanical and chemical hindrance into account in axon regeneration^34,35^. As illustrated in Fig. 7, SCI leads to a strong expression of CS56-positive CSPGs in the SCI group, while that is relatively weak in the MenSCs group. Thus, it can be concluded that MenSCs transplantation can inhibit glial scar after SCI.

The recognized hypothesis on the therapeutic effect of MSCs transplantation lies in the paracrine mechanism that MSCs secrete factors facilitate the recovery of injured tissue. BDNF is one of the most important growth factors in the neurotrophins family, playing a significant role in promoting neuronal survival and axonal regeneration^36,37^. Zhao et al. reported that the hybrid application of PRP and BDNF-overexpressing BMSCs can enhance rat’s spinal cord axonal remyelination in the hemi-section rat model^38^. Zemelko et al. used enzyme-linked immunosorbent assay (ELISA) and reported that the level of BDNF expressed in MenSCs was significantly higher than that in BMSCs and adipose-derived stem cells (ADSCs)^39,40^. The data obtained from immunostaining, qRT-PCR, and Western blot methods showed that the level of BDNF in the MenSCs group was higher than that of in the SCI group, especially at 2 weeks following MenSCs transplantation. However, when blocking BDNF-TrkB signaling through intrathecal administration of recombinant human TrkB-IgG, the promotion effect of locomotor function after MenSCs transplantation was inhibited significantly manifested by BBB score and the cell body area of motor neurons was significantly smaller demonstrated by Nissl staining at 14 days after surgery. We hypothesized that MenSCs transplantation might perform the therapeutic effect on the damaged spinal cord by increasing the level of BDNF in the spinal cord.

Inflammation is thought to be a key point in the secondary SCI^41^, involving the recruitment of chemokines, infiltration of monocytes and macrophages, and activation of local glial cells in injured spinal cord. TNF-α is an important inflammatory cytokine, containing several biological functions which plays a crucial role in immune inflammatory response^42^. TNF-α can generate neutrophils and monocyte chemokines and cause leukocyte adhesion and aggregation, thereby exacerbating the inflammatory response, causing scar formation as well as mediating secondary injury. Another important proinflammatory cytokine is IL-1β, which has a synergistic effect with TNF-α. Besides, IL-1β can induce leukocyte aggregation, and increase the secretion of adhesion molecules by endothelial cells^43^. The data achieved by the present study showed that the expression of TNF-α and IL-1β in the SCI group was significantly higher than that in sham group at 7, 14, and 28 days after SCI. Compared with the SCI group, the expression of TNF-α and IL-1β are considerably lower in the MenSCs group at each time point, indicating that MenSCs transplantation can reduce the expression of TNF-α and IL-1β in injured spinal cord, thereby decreasing inflammatory reaction.

Taken together, it can be concluded that MenSCs transplantation promotion of the functional recovery of SCI rats via enhanced expression and secretion of BDNF, reduced scar formation, and decreased the expression of inflammatory cytokine. So, the high basal level of BDNF synthesis in the MenSCs, along with noninvasive collection way and high proliferative rate make the MenSCs quite fit for SCI therapy. In future, it is required to evaluate the potential utility of MenSCs in clinical trial.

## Materials and methods

### Animal breeding

Adult female Sprague-Dawley rats (SD) were housed in Laboratory Animal Centre of Nantong University. Animal care and all experiment procedures were reviewed and approved by Jiangsu Institutes of Health Guide for the Care and Use of Laboratory Animals.

### Isolation and culture of human menstrual blood-derived stem cells (MenSCs)

The human MenSCs were isolated from female volunteers according to the protocol previously reported^14^. All the healthy women (*n* = 12) were on their second day during a menstrual cycle, and the menstrual blood specimens were collected with menstrual cups (Diva Cup Co., USA) inserted deeply into the vagina. The entire procedures were with consent of the volunteers and approved by the Ethics Committee of Nantong Maternal and Child Health Care Hospital, Affiliated to Nantong University, China. The Approval No. is 2016-023. Subsequently, the menstrual blood specimens were transferred into phosphate buffered saline (PBS) with mixed 1% penicillin/streptomycin at 4 °C for 24 h. Mononuclear cells were separated by a density gradient centrifugation with Ficoll-Paque (GE Healthcare™). The interlayer cells were collected and cultured with Dulbecco’s modified Eagle’s medium/nutrient mixture F12 (DMEM/F12) (GIBCO, USA) supplemented with 10% fetal bovine serum (FBS) (Life Technologies, USA) in a 10 cm dish (Corning Incorporated, USA). Cells were cultured with media changing every 2–3 days until adherent cells grew to 80–90% confluency. Then the cells were subcultured using 0.25% trypsin (Invitrogen, USA). The cells used in the experiments were at fourth to sixth passage.

### Identification of human MenSCs by flow cytometry

Flow cytometry was carried out to identify the CD surface antigen of the isolated MenSCs at passage 4–6 according to published protocols^14^. Briefly, about 10^5^ cells were resuspended in 100 mL PBS and incubated with primary antibodies CD146-APC, CD73-PerCP, CD105-PE, CD73-FITC (eBioscience), (1:100) at 4 ℃ for 1 h. Then, the cells were washed twice with PBS. The corresponding isotype antibodies (eBioscience) were set as negative controls. Cells were analyzed by a Flow Cytometer (FACS, Beckman Coulter).

### Induced differentiation of MenSCs to osteoblasts and adipocytes

For osteogenic differentiation, the MenSCs were plated at 3 × 10^3^ cells/cm^2^ and treated with the human MSC osteogenic differentiation medium (Cyagen, China). The osteogenic medium consisted of 100 nM dexamethasone (DEX), 0.2 mM ascorbate, 1 mM glutamine, 10 mM b-glycerophosphate, 1% penicillin–streptomycin, and 10% FBS and was completely changed every 3 days for up to 21 days. Cells were then fixed by 4% formaldehyde and stained with Alizarin red for 5 min.

For adipogenic differentiation, MenSCs were cultured in DMEM/F12 supplemented with 10% FBS, 1 μM DEX, 10 μg/ml recombinant human insulin, 0.5 mM 3-isobutyl-1-methyl-xanthine (IBMX) and 1 μM rosiglitazone (Sigma-Aldrich) for 6 days. Then, culture medium was replaced with DMEM/F12 supplemented with 10% FBS and cells were cultured for the next 3 days. Then they were treated with DMEM supplemented with 10% FBS, 1 μM DEX, 10 μg/ml recombinant human insulin, and 60 μM indomethacin (Sigma-Aldrich) up to 12 days^44^. For Oil Red O stain analysis, cells were fixed in 4% formaldehyde and then stained with Oil Red O for 30 min.

### SCI procedures

Adult female SD (220–250 g, Nantong University, *n* = 72) were randomly divided into three groups: Sham group (*n* = 24), SCI group (*n* = 24), and MenSCs group (*n* = 24). Rats were anesthetized via an intraperitoneal injection of 10% chloral hydrate (0.35 ml/kg). The spinous process and the vertebral lamina were removed to expose a circular region of dura at the T10 spinal level^45^. A longitudinal incision was made through the dura, exposing 0.5 cm of spinal cord. Incomplete SCI was made by microscissors as described previously^46^ (Fig. 2a–d). Then, DMEM/F12, MenSCs were implanted immediately using a microsyringe, and the muscles and skin were sutured in separate layers 10 min after injection. Postoperative care included regular bladder expression by manual abdominal pressure every 8 h until the bladder function recovered and intraperitoneally antibiotic treatment (Penicillin, 2 × 10^5^ IU/d for 7 days). Fresh spinal cord segment containing the injury epicenter and surrounding uninjured tissues (0.5 cm = 0.25 cm either side from the injury epicenter) were harvested immediately for WB and qRT-PCR.

### MenSCs transplantation

Ten minutes after SCI, 5 μl DMEM/F12 and MenSCs suspension (1 × 10^5^ cells/μl) were, respectively, injected into the injured site in both the SCI and MenSCs groups at 1 μg/min using a microsyringe (Fig. 2e, f). After surgery, the SCI group received 5 μl of culture medium (DMEM/F12), which was injected into the middle of the lesions, whereas the MenSCs group received 1.0 × 10^5^ MenSCs in 5 μl of culture medium. To maximize the engraftment of the MenSCs injected into the spinal cord, the needle was left in the spinal cord for 10 min after injection.

To trace exogenously introduced MenSCs in the injured site, MenSCs were stained with chloromethyl-benzamidodialkylcarbocyanine (CM-Dil; Molecular Probes, USA). Briefly, MenSCs in suspension were washed with PBS and incubated with CM-Dil at a concentration of 2 mg/ml PBS for 5 min at 37 °C and 15 min at 4 °C. After labeling, CM-Dil-labeled MenSCs were washed three times with culture medium and applied to in vivo assay.

### Intrathecal catheter operation

The intrathecal catheter operation was performed 7 days before SCI. Adult female SD (220–250 g, Nantong University, *n* = 50) were randomly divided into five groups: Sham group (*n* = 10), SCI group (*n* = 10), MenSCs group (*n* = 10), SCI/TrkB-IgG group (*n* = 10), and MenSCs/TrkB-IgG group (*n* = 10). Briefly, all rats were anesthetized via an intraperitoneal injection of 10% chloral hydrate (0.35 ml/kg). A 6 cm polyethylene PE-10 catheter (Inner diameter [ID]: 0.28 mm; outer diameter [OD]: 0.61 mm, Smiths Medical International Ltd., UK) was sterilized and filled with PBS, then inserted about 2 cm between 3rd and 4th lumbar vertebra, therefore, the end of the catheter corresponds to T10 spinal cord level. After the intrathecal catheter, 20 µl of phosphate-buffered saline (PBS) was used to irrigate the catheter by a 25 µl microsyringe (Model 1702, Hamilton company, GR, Switzerland), and then closed the catheter immediately. All rats were housed one per cage after intrathecal catheter. Antibiotics (Penicillin, 2 × 10^6^ IU/rat, i.m.) were given one time per day for 3 consecutive days. On the 7th day after operation, 20 µl of 2% lidocaine hydrochloride and then 20 µl of PBS were injected along the catheter using a 25 µl microsyringe, and the catheter was immediately closed. All the rats showed paralysis on both hindlimbs and tail, suggesting that the catheter was in place.

After 7 days of intrathecal catheter, all rats were analgesic and anesthetized as above. Then, SCI procedures were performed.

### Intrathecal administration of TrkB-IgG

All Alzet Osmotic pumps (model 2002, Alzet, Cupertino, CA) needed to be incubated with saline for 12 h before inserted. Recombinant human TrkB-Fc chimera (TrkB-IgG, R&D Systems, Minneapolis, USA) was used to block BDNF-TrkB signaling. After SCI, PBS alone or TrkB-IgG dissolved in PBS (0.25 µg/µl) was filled into minipumps^47^. A 1 cm PE-50 (ID: 0.58 mm; OD: 0.96 mm; Smiths Medical International Ltd., UK) attached the pumps and the PE-10 catheter inserted before. Prior to attachment, 20 µl PBS was injected through the catheter using a 25 µl microsyringe to observe catheter patency. Finally, the pump and polyethylene catheter were placed subcutaneously on the back of rats. The TrkB-IgG was pumped continually at a rate of 3 µg/day for 4 weeks. Since the TrkB-IgG had a 2-week in vivo activity^48^, the same procedure was performed to replace the pumps after 3 weeks of all groups. Antibiotics (Penicillin, 2 × 10^6^ IU/rat, i.m.) were given as above.

### BBB score

The 21 point BBB locomotion scale was used to assess locomotor recovery^49^. The rats were placed in an open field (80 × 130 × 30 cm^3^) and were observed individually for 5 min by two observers who were blinded to the allocation of the animals. The rats were tested at 0, 1, 3, 5, 7, 14, 21, and 28 days after SCI surgery.

### Nissl staining

For Nissl staining, spinal cord sections were stained with cresyl violet, dehydrated through graded alcohols (70%, 95%, 100% 2×), placed in xylene and coverslipped using DPX mountant. The morphology of the spinal cord was acquired on a Zeiss Axio Scope A1 fluorescence microscope and the cell body area of motor neurons was analyzed using Image Pro Plus (Media Cybernetics LP, Maryland, USA).

### Immunohistochemistry

Rats were anesthetized and perfused through the heart with 4% paraformaldehyde. The spinal cords were removed from the vertebral columns, post-fixed in the same fixative, and stored in 30% sucrose at 4 °C. 2-cm-long horizontal sections of spinal cords containing the lesion site were sectioned on a cryostat, and 25 μm coronal sections were collected. Tissue sections were incubated in blocking solution consisting of 3% normal donkey serum (NDS, Jackson) and 0.3% Triton X-100 (Sigma-Aldrich, St. Louis) in PBS at room temperature (RT) for 30 min and then reacted with specific primary antibodies BDNF (Abcam; 1:1000), MAP2 (Millipore; 1:1000), NF200 (Abcam; 1:1000), CS56 (Abcam; 1:500) at 4 °C overnight. Each sample was washed with PBS three times, for 10 min each, and then stained by secondary antibodies include Goat anti-rabbit, Alexa Fluor^®^ 488, Goat anti-mouse, Alexa Fluor^®^ 488 (invitrogen; 1:1000) for 1 h. Finally, cell nuclei were stained with Hoechst 33342 (Sigma; 1:1000). Images were acquired on a fluorescence microscope (Olympus BX 51, Tokyo, Japan). Image-Pro Plus 6.0 was used for quantitative analysis. During image acquisition, the illumination level of each imaging session was maintained by stabilizing the light source, and the settings of the camera and the lamp were constant.

For haematoxylin and eosin (H&E) staining, the spinal cords samples were embedded in paraffin, and 5-μm sections in the sagittal plane were prepared. Paraffin-embedded sections were stained with H&E.

### Quantitative analyses

The cavity volume was calculated from H&E-stained sections of three rats from each group. Briefly, the areas of cavities were measured on sections using Image-Pro Plus software and multiplied by the thickness of the section (5 μm for each section). The spinal cord volume at the lesion site was measured as follows: the average diameter of the spinal cord at the lesion site was obtained from three levels: at the epicenter of the lesion, at the level 0.5 cm rostral to and at the level 0.5 cm caudal to the epicenter. The area of the cross-section of the spinal cord at the lesion site was calculated using the average diameter. The volume was calculated by multiplying this cross-section by 1 cm (the length of the spinal cord). The relative cavity volume (%) was calculated by dividing the cavity volume by the spinal cord volume at the lesion site.

For quantification the number of MAP2-positive neurons and NF-200-positive axons, three areas (0.1 mm^2^) at the lesion center were randomly selected from each rat. The number of axons or neurons in each group was calculated. For quantification of CS-56 and BDNF IHC images, Image-Pro Plus software were used to carry out the MOD of CS-56-positive and BDNF-positive action at the lesion center.

### Quantitative reverse transcription-polymerase chain reaction

For qRT-PCR, total RNA was reverse-transcribed using PrimeScriptTMRT Master Mix (Takara; RR036A). All reactions were performed using SYBR Premix Ex Taq (Tli RNaseH Plus); (Takara; DRR420A). Primers were designed using Primer Express software (Applied Biosystems) and experimentally validated. the sequences for primers used are as follows:

**Table:** 

Gene	qRT-PCR forward primer	qRT-PCR reverse primer
BDNF	GGTCACAGTCCTGGAGAAAG	GTCTATCCTTATGAACCGCC
TNF-α	TGTCTGTGCCTCAGCCTCTTC	TTTGGGAACTTCTCCTCCTTGT
IL-1β	ACTCATTGTGGCTGTGGAGA	ACACACTAGCAGGTCGTCAT
GAPDH	CCTCAAGATTGTCAGCAAT	CCATCCACAGTCTTCTGAGT

The expression of each gene was defined from the threshold cycle (*C*_t_), and relative expression levels were calculated by using the _△△_CT method after normalization with reference to expression of the housekeeping gene GAPDH. Results are means from three individual experiments.

### Western blotting

Protein samples were prepared from spinal cords of each group. Protein concentrations were measured by the BCA protein assay kit (Thermo Scientific), and cell lysates were applied to 8% SDS–polyacrylamide gels (Bio-Rad), transferred to a PVDF membrane (Millipore). Primary antibodies were β-actin (Sigma), BDNF (Abcam), β-actin was included as a loading control.

### Statistical analysis

All data were presented as mean ± SEM values, and were analyzed by two-way ANOVA repeated measurement followed by Bonferroni post hoc test for statistical significance between groups using SPSS Statistics 22.0 (SPSS Inc., Chicago, IL, USA), with significance measured at *P < 0.05, **P < 0.01, or ***P < 0.001.
